# A recessive homozygous p.Asp92Gly *SDHD* mutation causes prenatal cardiomyopathy and a severe mitochondrial complex II deficiency

**DOI:** 10.1007/s00439-015-1568-z

**Published:** 2015-05-26

**Authors:** Charlotte L. Alston, Camilla Ceccatelli Berti, Emma L. Blakely, Monika Oláhová, Langping He, Colin J. McMahon, Simon E. Olpin, Iain P. Hargreaves, Cecilia Nolli, Robert McFarland, Paola Goffrini, Maureen J. O’Sullivan, Robert W. Taylor

**Affiliations:** Wellcome Trust Centre for Mitochondrial Research, Institute of Neuroscience, The Medical School, Newcastle University, Newcastle upon Tyne, NE2 4HH UK; Department of Life Sciences, University of Parma, Parma, Italy; Children’s Heart Centre, Our Lady’s Children’s Hospital, Crumlin, Dublin, Ireland; Department of Clinical Chemistry, Sheffield Children’s Hospital, Sheffield, UK; Neurometabolic Unit, National Hospital for Neurology and Neurosurgery, London, UK; Department of Pathology, Our Lady’s Children’s Hospital, Crumlin, Dublin, Ireland

## Abstract

Succinate dehydrogenase (SDH) is a crucial metabolic enzyme complex that is involved in ATP production, playing roles in both the tricarboxylic cycle and the mitochondrial respiratory chain (complex II). Isolated complex II deficiency is one of the rarest oxidative phosphorylation disorders with mutations described in three structural subunits and one of the assembly factors; just one case is attributed to recessively inherited *SDHD* mutations. We report the pathological, biochemical, histochemical and molecular genetic investigations of a male neonate who had left ventricular hypertrophy detected on antenatal scan and died on day one of life. Subsequent postmortem examination confirmed hypertrophic cardiomyopathy with left ventricular non-compaction. Biochemical analysis of his skeletal muscle biopsy revealed evidence of a severe isolated complex II deficiency and candidate gene sequencing revealed a novel homozygous c.275A>G, p.(Asp92Gly) *SDHD* mutation which was shown to be recessively inherited through segregation studies. The affected amino acid has been reported as a Dutch founder mutation p.(Asp92Tyr) in families with hereditary head and neck paraganglioma. By introducing both mutations into *Saccharomyces cerevisiae,* we were able to confirm that the p.(Asp92Gly) mutation causes a more severe oxidative growth phenotype than the p.(Asp92Tyr) mutant, and provides functional evidence to support the pathogenicity of the patient’s *SDHD* mutation. This is only the second case of mitochondrial complex II deficiency due to inherited *SDHD* mutations and highlights the importance of sequencing all *SDH* genes in patients with biochemical and histochemical evidence of isolated mitochondrial complex II deficiency.

## Introduction

Mitochondrial respiratory chain disease arises from defective oxidative phosphorylation (OXPHOS) and represents a common cause of metabolic disease with an estimated prevalence of 1:4300 (Gorman et al. 2015; Skladal et al. 2003). Under aerobic conditions, metabolised glucose, fatty acids and ketones are the OXPHOS substrates, shuttling electrons along the respiratory chain whilst concomitantly creating a proton gradient by actively transporting protons across the mitochondrial membrane. The resultant proton gradient is exploited by ATP synthase to drive ATP production. Under anaerobic conditions, for example where atmospheric oxygen is scarce or during periods of exertion, ATP synthesis is produced primarily during glycolysis (Horscroft and Murray 2014).

The mitoproteome consists of an estimated 1400 proteins (Pagliarini et al. 2008), including the 13 polypeptides and 24 non-coding tRNA and rRNA genes encoded by the mitochondria’s own genetic material (mtDNA) that are exclusively maternally transmitted. The remaining genes of the mitoproteome are located on either the autosomes or sex chromosomes and as such are transmitted from parent to child in a Mendelian fashion. Defects in a number of mtDNA and nuclear-encoded genes have been linked to human disease, often associated with a vast genetic and clinical heterogeneity and further compounded by few genotype–phenotype correlations which help guide molecular genetic investigations.

Succinate dehydrogenase is a crucial metabolic enzyme complex that is involved in both the Krebs cycle and the mitochondrial respiratory chain. It is composed of two catalytic subunits (the flavoprotein SDHA, and Fe–S-containing SDHB) anchored to the inner mitochondrial membrane by the SDHC and SDHD subunits. All four subunits and the two known assembly factors are encoded by autosomal genes (*SDHA*, *SDHB*, *SDHC*, *SDHD*, *SDHAF1* and *SDHAF2*, hereafter referred to as *SDHx*). Congenital recessive defects involving *SDHx* genes are associated with diverse clinical presentations, including leukodystrophy and cardiomyopathy (Alston et al. 2012).

A recent review describes *SDHA* mutations as the most common cause of isolated complex II deficiency, with 16 unique mutations reported in 30 patients (Ma et al. 2014; Renkema et al. 2014); the next most common cause are mutations in *SDHAF1*, 4 mutations have been reported in 13 patients (Ghezzi et al. 2009; Ohlenbusch et al. 2012). Just one mitochondrial disease patient is reported to harbour either *SDHB* (Alston et al. 2012) or *SDHD* (Jackson et al. 2014) mutations and metabolic presentations have yet to be reported in association with *SDHC* or *SDHAF2*.

In addition to their role in primary respiratory chain disease, SDHx mutations can act as drivers of neoplastic transformation following loss of heterozygosity (LOH). One of the most common causes of head and neck paraganglioma (HNPGL) is LOH at the SDHD locus. These mutations are inherited in a dominant manner with a parent of origin effect; typically only paternally inherited SDHD mutations are associated with HNPGL development (Hensen et al. 2011).

Here, we report a neonate who presented prenatally with cardiomyopathy due to a novel homozygous *SDHD* mutation. This is the second report of recessive *SDHD* mutations resulting in a primary mitochondrial disease presentation and serves to characterise the biochemical, histochemical and functional consequences of our patient’s molecular genetic defect. Moreover, the affected amino acid, p.Asp92, has been reported as a Dutch founder mutation in families with hereditary PGL, albeit the substituted residue differs. We have used the yeast, *Saccharomyces cerevisiae,* which has proven to be a useful model system to study the effects of SDHx gene mutations (Goffrini et al. 2009; Panizza et al. 2013), to provide functional evidence supporting the pathogenicity of the *SDHD* mutation identified in our patient and, to a lesser extent, that of the PGL-associated p.Asp92Tyr mutation.

## Patient and methods

**Fig. 1:**
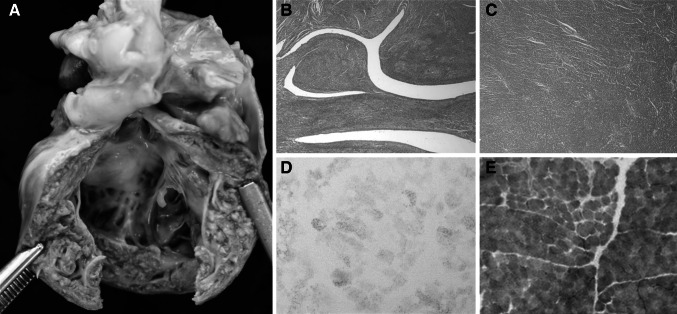
Macroscopic and microscopic analysis of patient cardiac and skeletal muscle. **a** Macroscopic examination of patient heart revealed obvious non-compaction of the hypertrophic left ventricle. **b** Microscopic assessment of left ventricle confirms non-compaction compared to neonatal control tissue (**c)**. Histochemical assessment of patient muscle biopsy shows a marked decrease in the activity of succinate dehydrogenase activity (**d**) compared with a control muscle **(e)**

The patient is the third child born to unrelated Irish parents. Foetal heart abnormalities were identified on an anomaly scan at 31-weeks gestation, which prompted foetal echocardiography. A normally situated heart with normal systemic and pulmonary venous drainage was reported. Right to left shunting was noted at the patent foramen ovale and ductus arteriosus, consistent with gestational age. The left ventricle and left atrium were severely dilated with moderate–severe mitral regurgitation. There was severe left ventricular systolic dysfunction, but no evidence of pericardial effusion or ascites. Rhythm was normal sinus with a foetal heart rate between 100 and 120 beats per minute and subsequent weekly foetal echocardiogram showed no further progression of cardiac dysfunction or development of hydrops. Cardiac MRI at 32-weeks gestation showed marked left ventricular hypertrophy and dilation. A clinical diagnosis of dilated cardiomyopathy was considered and the parents were counselled that the prognosis for postpartum survival was poor. The proband was born by elective caesarean section at 37 + 6 weeks gestation with a birth weight of 2620 g (9th–25th centile) and occipital circumference of 34.5 cm (50th–75th centile). He had no dysmorphic features. He was transferred to neonatal intensive care on 100 % oxygen to maintain his saturations in the low 90 s. An additional heart sound and loud murmur were noted, along with hepatomegaly (4 cm below costal margin) but without splenomegaly. By 12 h of age, his condition had deteriorated significantly; echocardiogram showed dilation of the inferior vena cava, hepatic veins, right atrium and interatrial septal bowing. He had moderate tricuspid regurgitation and very poor biventricular function with non-compaction hypertrophy. The proband died the following evening following withdrawal of life support with parental consent. At postmortem examination, the heart weighed 43 g (normal = 13.9 ± 5.8 g). The right atrium was particularly enlarged. The endocardium of the right atrium but particularly also the right ventricle showed fibroelastosis. The right ventricle was remarkably diminutive and underdeveloped. The right atrium had a 7-mm-diameter patent foramen ovale. There was obvious non-compaction of the hypertrophic left ventricular myocardium (Fig. 1). Cardiac muscle, skeletal muscle and a skin biopsy were referred for laboratory investigations. Informed consent was obtained from the parents for the clinical and laboratory investigations and publication of the results.

## Histochemical and biochemical assessment of metabolic function

Histological and histochemical assay of 10 μm serial sections of patient muscle biopsy was performed according to standard protocols. The measurement of respiratory chain enzyme activities was determined spectrophotometrically as described previously (Kirby et al. 2007). Fibroblast culture and measurement of β-oxidation flux in cultured fibroblasts using [9, 10-^3^H] myristate, [9, 10-^3^H]palmitate and [9, 10-^3^H]oleate was performed as described elsewhere (Manning et al. 1990; Olpin et al. 1992, 1997).

## Cytogenetic and molecular genetic investigations

Karyotyping of cultured fibroblasts and DNA extraction from patient muscle were performed according to standard protocols. Primers were designed to amplify each coding exon, plus intron–exon boundaries, of the *SDHA*, *SDHB*, *SDHC*, *SDHD*, *SDHAF1* and *SDHAF2* genes. PCR amplicons were Sanger sequenced using BigDye3.1 chemistry (Applied Biosystems) and capillary electrophoresed on an ABI3130xl bioanalyser (Applied Biosystems) using standard methodologies. Resultant sequencing chromatograms were compared to the Genbank reference sequences: *SDHA* (NM_004168.2), *SDHB* (NM_003000.2), *SDHC* (NM_003001.3), *SDHD* (NM_003002.3), *SDHAF1* (NM_001042631.2) and *SDHAF2* (NM_017841.2). All gene variants were annotated using dbSNP build 138 whilst ESP6500 and 10 k genome project data allowed determination of allele frequencies. Parental DNA samples were screened to investigate allele transmission.

## In silico pathogenicity prediction tools and structural modelling

The effect of the p.(Asp92Gly) substitution on SDHD function was predicted using the in silico tools SIFT (Ng and Henikoff 2003), Align GVGD (Tavtigian et al. 2006) and Polyphen (Adzhubei et al. 2010), all running recommended parameters. To determine whether the tertiary structure of the protein was affected by the mutation, the wild-type (NP_002993) and mutant SDHD protein sequences were input to PSIPRED (Jones 1999) and I-TASSER (Yang et al. 2014); I-TASSER output was visualised using UCSF Chimera (Pettersen et al. 2004).

## Blue native polyacrylamide gel electrophoresis (BN-PAGE)

Mitochondria-enriched pellets were obtained from 15 mg skeletal muscle as previously described (Nijtmans et al. 2002) and solubilised in 1× sample buffer (Invitrogen, BN20032), 2 % dodecyl-β-d-maltoside (Sigma) and 5 % glycerol. Mitochondria were pelleted for 15 min at 100,000 g. Protein concentrations were determined by Pierce™ BCA Protein Assay Kit according to manufacturer’s protocol (ThermoScientific). Protein samples (25 μg) were separated on NativePAGE™ Novex^®^ 4–16 % Bis–Tris Protein Gels (Sigma) then transferred onto a PVDF membrane. Immunodetection of assembled respiratory chain complexes was performed using primary monoclonal antibodies (mitosciences) raised against complex-specific proteins: Complex I (NDUFB8, Abcam, ab110242), Complex II (SDHA, MitoSciences, MS204), Complex III (CORE2 Abcam, ab14745), Complex IV (COX1 Abcam, ab14705) and Complex V (ATP5A Abcam, ab14748). Following HRP-conjugated secondary antibody application (Dako), detection was undertaken using the ECL^®^ plus chemiluminescence reagent (GE Healthcare Life Sciences, Buckinghamshire, UK) and a ChemiDoc MP imager (Bio-Rad Laboratories).

## Western blot

Mitochondria-enriched pellets prepared as above were lysed on ice in 50 mM Tris pH 7.5, 130 mM NaCl, 2 mM MgCl_2_, 1 mM PMSF and 1 % NP-40. Protein concentration was calculated using the Bradford method (Bradford 1976). 13 µg of enriched mitochondrial proteins was loaded on a 12 % sodium dodecyl sulphate polyacrylamide gel with 1× dissociation buffer, electrophoretically separated and subsequently transferred onto a PVDF membrane. Immunodetection was performed using primary antibodies raised against complex II SDHA (MitoSciences, MS204) and SDHD (Merck Millipore, ABT110) and a mitochondrial marker protein, Porin (Abcam, ab14734). Following secondary antibody application (Dako), detection was undertaken using the ECL^®^ plus chemiluminescence reagent (GE Healthcare Life Sciences, Buckinghamshire, UK) and ChemiDoc MP imager (Bio-Rad Laboratories).

## Yeast strains and media

Yeast strains used in this study were BY4741 (*MATa*; *his3∆1 leu2∆0 met15∆0 ura3∆0*) and its isogenic sdh4:kanMX4. Cells were cultured in yeast nitrogen base (YNB) medium: 0.67 % yeast nitrogen base without amino acids (ForMediumTM), supplemented with 1 g/l of drop-out powder (Kaiser et al. 1994) containing all amino acids and bases, except those required for plasmid maintenance. Various carbon sources (Carlo Erba Reagents) were added at the indicated concentration. For the respiration and mitochondria extraction, cells were grown to late-log phase in the YNB medium supplemented with 0.6 % glucose. Media were solidified with 20 g/l agar (ForMediumTM) and strains were incubated at 28 or 37 °C.

## Construction of yeast mutant alleles

The *sdh4*Asp98Gly and *sdh4*Asp98Tyr mutant alleles were obtained by site-directed mutagenesis using the overlap extension technique (Ho et al. 1989). In the first set of PCR reactions, the *SDH4* region was obtained using the forward primer ESDH4F and the following reverse mutagenic primer sdh4R98G 5′-CATGACAGAAAAGAAAGA**ACC**AGCTGCAGTGGATAACGGAC-3′ and sdh4R98Y 5′-CATGACAGAAAAGAAAGA**GTA**AGCTGCAGTGGATAACGGAC-3′ where base changes are indicated in bold. The second *SDH4* region was obtained using the forward mutagenic primer sdh4F98G and sdh4F98Y, complementary to sdh4R98G and sdh4R98Y, and the reverse primer XSDH4R. The final mutagenized products were obtained using the overlapping PCR fragments as template with ESDH4F and XSDH4R as external primers. The products were then digested with *Eco*RI and *Xba*I and cloned in *Eco*RI–*Xba*I-digested pFL38 centromeric plasmid (Bonneaud et al. 1991). The mutagenized inserts were verified by sequencing and the pFL38 plasmid-borne *SDH4* and *SDH4* mutant alleles were transformed in the BY4741 using the lithium-acetate method (Gietz and Schiestl 2007).

## Isolation of mitochondria, enzyme assay and respiration

Oxygen uptake was measured at 28 °C using a Clark-type oxygen electrode in a 1-ml stirred chamber containing 1 ml of air-saturated respiration buffer (0.1 M phthalate–KOH, pH 5.0) and 10 mM glucose (Oxygraph System, Hansatech Instruments, England). The reaction was initiated with the addition of 20 mg of wet weight of cells, as previously described (Goffrini et al. 2009). Preparation of mitochondria and succinate dehydrogenase DCPIP assay was conducted as described (Goffrini et al. 2009). The succinate:decylubiquinone DCPIP reductase assay was conducted as previously described (Jarreta et al. 2000; Oyedotun and Lemire 2001). Protein concentration was determined by the Bradford method using the Bio-Rad protein assay following the manufacturer’s instructions (Bradford 1976).

## Results

### Pathological, histochemical and biochemical analysis

Histopathological examination of the patient’s heart revealed non-compaction of the left ventricular myocardium (Fig. 1a, b). Histological investigations reported normal skeletal muscle morphology whilst histochemical analysis of fresh-frozen muscle biopsy sections revealed a global reduction of succinate dehydrogenase (complex II) activity compared to aged-matched control samples (Fig. 1d, e). Spectrophotometric analysis of respiratory chain function in patient muscle homogenate revealed a marked defect in complex II activity (patient 0.042 nmols/min/unit citrate synthase activity; controls 0.145 ± 0.047 nmols/min/unit citrate synthase activity (*n* = 25) representing ~30 % residual enzyme activity; the activities of complex I, complex III and complex IV were all normal (not shown). Fatty acid oxidation flux studies on cultured fibroblasts gave normal results which excluded virtually all primary defects of long- and medium-chain fatty acid oxidation as the cause of the underlying cardiac pathology. There was no evidence of an underlying aminoacidopathy and serum urea and electrolytes were within normal limits. Investigations of glucose and lactate levels were not performed. The monolysocardiolipin/cardiolipin (ML/CL) ratio on a postmortem sample was 0.03 and the neutrophil count was within normal limits.

### Cytogenetic and molecular genetic investigations

**Fig. 2:**
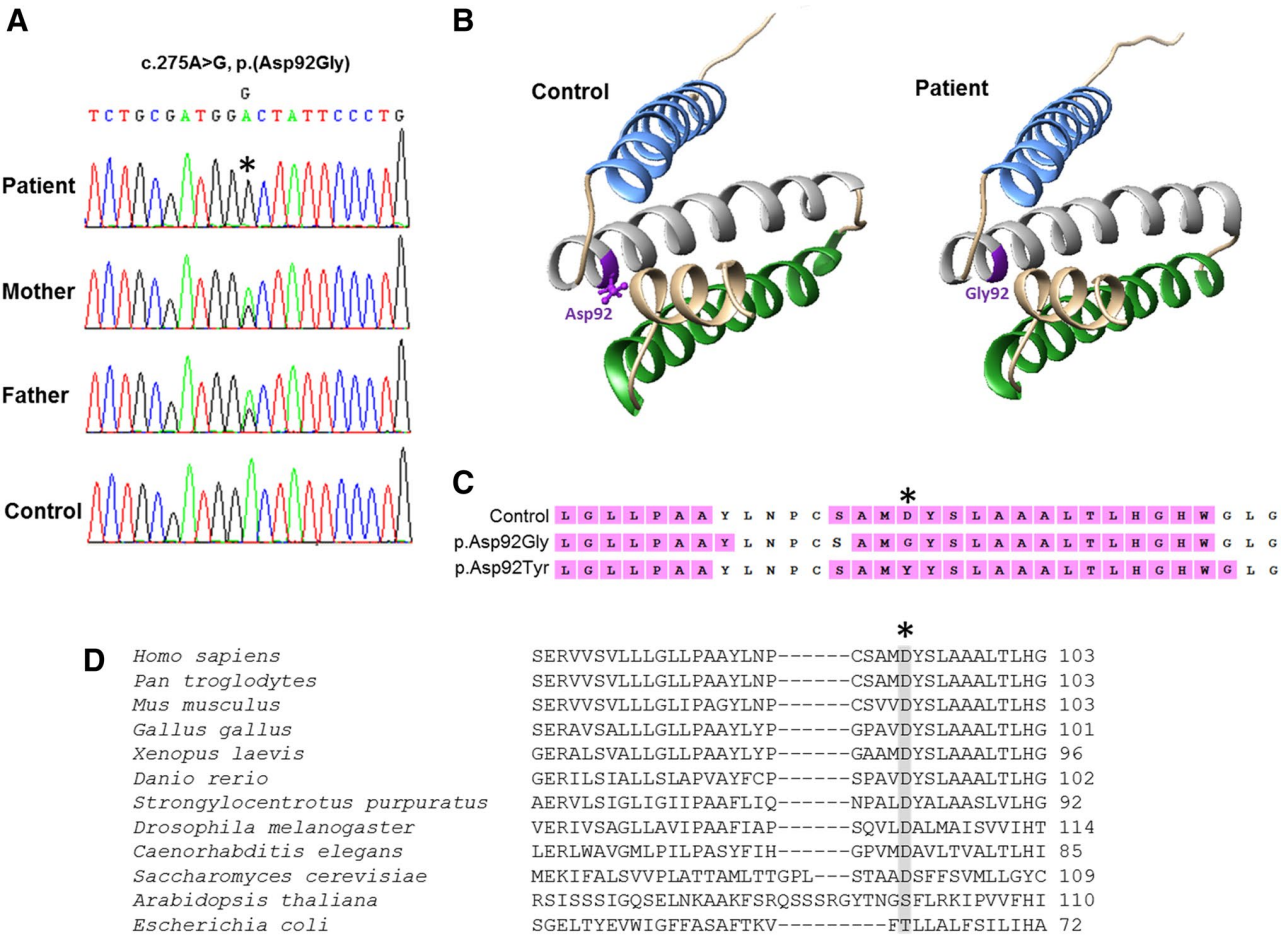
Molecular genetic and in silico investigations. **a** Identification of a pathogenic *SDHD* mutation. A homozygous c.275A>G, p.(Asp92Gly) *SDHD* mutation was identified in the proband, with parental DNA screening supporting recessive inheritance. The mutation affects a highly conserved p.Asp92 residue in the *SDHD*-encoded subunit of succinate dehydrogenase (SDH). **b** Structural modelling. I-TASSER prediction of control and patient SDHD tertiary structure shows the p.Asp92 residue located within a transmembrane helix domain and the p.Asp92Gly substitution is predicted to have little impact on SDHD tertiary structure. **c** PSIPRED output predicts minor alterations to two of the SDHD helices from the patient p.(Asp92Gly) and HNPGL p.(Asp92Tyr) substitutions compared to control sequence. Predicted helix residues shown in *pink*; unshaded residues are located in coil domains. **d** Multiple sequence alignment of this region of the SDHD subunit was performed using ClustalW and confirms that the p.(Asp92Gly) mutation affects an evolutionary conserved residue (*shaded*). Alignments were manually corrected on the basis of the pairwise alignment obtained with PSI-BLAST

Karyotyping reported a normal 46 XY profile, consistent with no large genomic rearrangements. Following identification of an isolated complex II deficiency, Sanger sequencing of all six *SDHx* genes was undertaken and a novel homozygous c.275A>G, p.(Asp92Gly) variant was identified in *SDHD* (ClinVar Reference ID: SCV000196921). Results from parental carrier testing were consistent with an autosomal recessive inheritance pattern, with each parent harbouring a heterozygous c.275A>G, p.(Asp92Gly) *SDHD* variant (Fig. 2a). The p.Asp92 *SDHD* residue is highly conserved (Fig. 2d) and the c.275A>G variant is not reported on either the ESP6500 or 1KGP suggesting that it is rare in the general population. Whilst the c.275A>G, p.(Asp92Gly) *SDHD* variant has not been previously reported, another mutation affecting the same residue—c.274G>T, p.(Asp92Tyr)—has been reported in association with familial PGL and PCC (Hensen et al. 2011). In silico predictions were strongly supportive of a deleterious effect; 100 % sensitivity and 100 % specificity were reported by SIFT and polyphen for both the p.(Asp92Gly) and p.(Asp92Tyr) variants. Both variants were assigned an aGVGD class of C65 (highly likely to be detrimental to protein function)—the p.(Asp92Gly) variant was reported to have a grantham difference (GD) value of 93.77, whilst the GD for the p.(Asp92Tyr) variant was 159.94 (GD > 70 is associated with C55/C65 variant classes). SDHD tertiary structure was not predicted to be markedly impacted by the patient’s p.(Asp92Gly) substitution; no gross conformational change was reported by I-TASSER (Fig. 2b), whilst PSIPRED predicted only a mild alteration to the helix structure (Fig. 2c).

### Functional effect of the c.275A>G, p.(Asp92Gly) SDHD mutation on protein expression and complex assembly

**Fig. 3:**
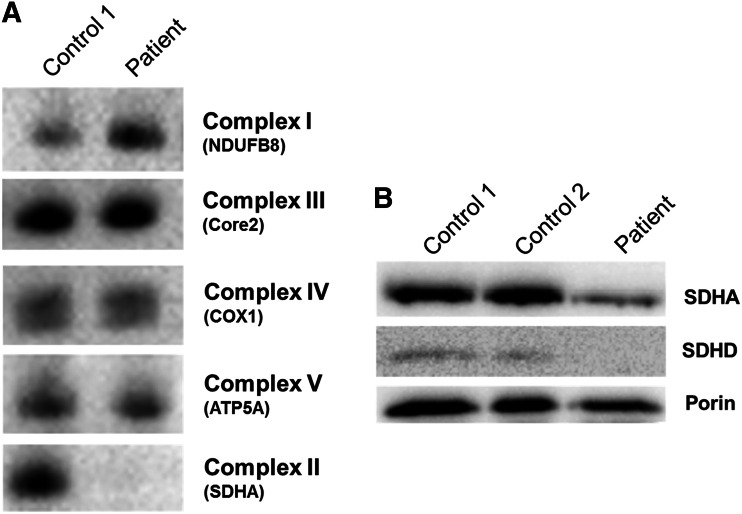
Investigation of OXPHOS complex activities and protein expression in patient and controls. **a** BN-PAGE analysis of mitochondria isolated from patient and control muscle homogenates revealed a reduction of assembled complex II in patient muscle with normal assembly of all other OXPHOS complexes. **b** SDS-PAGE analysis of patient and control proteins probed with antibodies against Porin (a loading control) and the SDHA and SDHD subunits of succinate dehydrogenase revealed a stark reduction in SDH steady-state protein levels in patient muscle, consistent with subunit degradation thereby supporting the pathogenicity of the p.(Asp92Gly) variant

Having identified an excellent candidate mutation, assessment of respiratory chain complex assembly by one-dimensional BN-PAGE revealed a marked decrease in fully assembled Complex II, whilst levels of fully assembled complexes I, III, IV and V were comparable to controls (Fig. 3a). Western blot of mitochondrial proteins in patient muscle was performed which confirmed a significant reduction of the SDHD and SDHA proteins compared to both equally loaded control muscle samples and Porin, a mitochondrial marker protein (Fig. 3b).

### Functional studies in a yeast model

To further assess the pathogenicity of the patient’s novel p.(Asp92Gly) *SDHD* variant, we performed complementation studies using a strain of *S. cerevisiae* lacking the *SDH4* gene hereafter referred to as Δ*sdh4*. The *SDH4* gene is the yeast orthologue of human *SDHD* and although the human and yeast protein have a low degree of conservation (16 % identity and 36 % similarity) the p.Asp92 residue is conserved between the two species, corresponding to p.Asp98 in yeast (Fig. 2d). We then introduced the change equivalent to the human p.(Asp92Gly) variant into the yeast *SDH4* wild-type gene cloned in a centromeric vector thus obtaining the *sdh4*^D98G^ mutant allele. Since another mutation involving the same residue, p.(Asp92Tyr), has been reported as a cause of paraganglioma, a second mutant allele, *sdh4*^D98Y^, was also constructed to compare the phenotype between the two different amino acid substitutions. The *SDH4*, *sdh4*^D98G^ and *sdh4*^D98Y^ constructs and the empty plasmid pFL38 were then transformed into the Δ*sdh4* strain. To test the possible effects on mitochondrial function, we first evaluated the oxidative growth by spot assay analysis on mineral medium supplemented with either glucose or ethanol, at 28 and 37 °C.

**Fig. 4:**
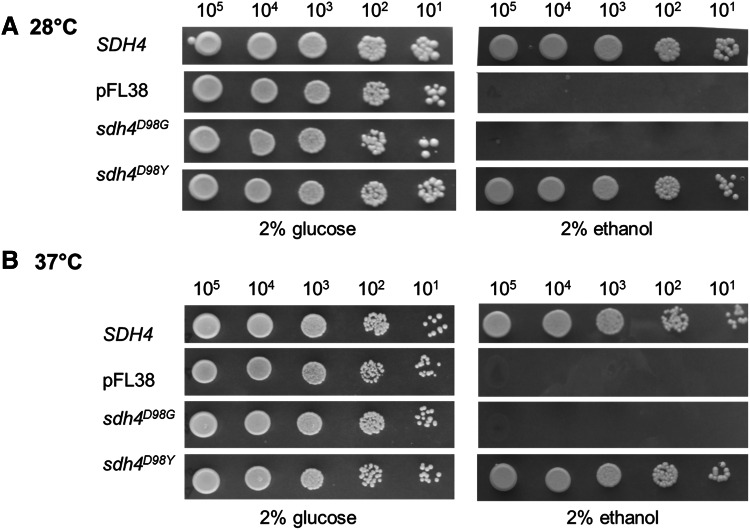
Oxidative growth phenotype in yeast. The strain BY4741 ∆*sdh4* was transformed with a pFL38 plasmid carrying either the wild-type *SDH4*, the empty vector or the mutant alleles *sdh4* ^D98G^ and *sdh4* ^D98Y^. Equal amounts of serially diluted cells from exponentially grown cultures (10^5^, 10^4^, 10^3^, 10^2^, 10^1^) were spotted onto yeast nitrogen base (YNB) plates supplemented with either 2 % glucose or 2 % ethanol. The growth was scored after 3-day incubation at 28 °C (**a**) and 37 °C (**b**)

**Fig. 5:**
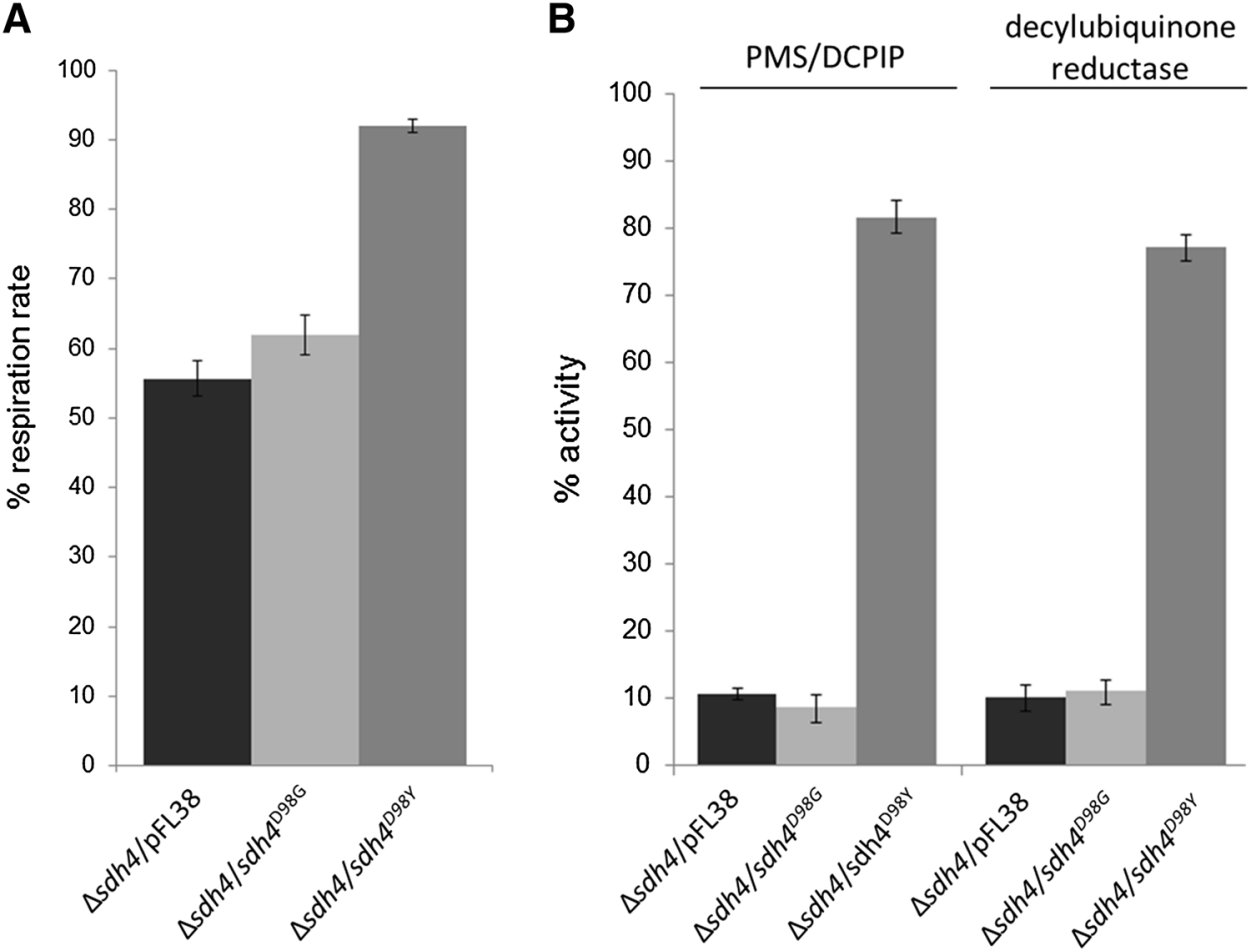
**a** Oxygen consumption rates. Respiration was measured in cells grown in YNB supplemented with 0.6 % glucose at 28 °C. The values observed for the *sdh4* mutant cells are reported as a percentage of the wild-type *SDH4* cell respiratory rate, 40.46 ± 1.54 nmol min^−1^ mg^−1^. **b** Complex II activity. PMS/DCPIP reductase and decylubiquinone reductase activities were measured in mitochondria extracted from cells grown exponentially at 28 °C in YNB supplemented with 0.6 % glucose. The values of the *sdh4* mutants are expressed as percentage of the activities obtained in the wild-type strain

A clear growth defect was observed for the Δ*sdh4/sdh4*^*D98G*^ strain in ethanol-containing plates incubated both at 28 and 37 °C (Fig. 4a), with growth similar to that of the *sdh4* null mutant. Contrariwise, the Δ*sdh4/sdh4*^D98Y^ strain did not exhibit an OXPHOS-deficient phenotype at either temperature tested (Fig. 4b) or in either oxidative carbon source analysed (not shown). To further investigate the OXPHOS defect, oxygen consumption and SDH activity were measured. The oxygen consumption rate of the Δ*sdh4*/*sdh4*^D98G^ mutant was 55 % less than that of the parental strain Δsdh4/SDH4 (Fig. 5a), likewise, succinate dehydrogenase enzyme activities (PMS/DCPIP reductase and decylubiquinone reductase) were both severely reduced, with levels similar to those of the null mutant (Fig. 5b). Consistent with the results obtained from growth experiments the oxygen consumption rate of the Δ*sdh4*/*sdh4*^D98Y^ mutant was not impaired (Fig. 5a) but both SDH activities (PMS/DCPIP reductase and decylubiquinone reductase) were partially reduced (80 and 75 % residual activity) in Δ*sdh4*/*sdh4*^D98Y^ mitochondria (Fig. 5b). Together, these data support the pathogenicity of our patient’s novel p.(Asp92Gly) *SDHD* variant.

## Discussion

Mitochondrial complex II deficiency is one of the rarest disorders of the OXPHOS system, accounting for between 2 and 8 % of mitochondrial disease cases (Ghezzi et al. 2009; Parfait et al. 2000) with only ~45 cases reported in the literature. We report a newborn boy presenting with left ventricular hypertrophy on foetal ultrasound at 32-weeks gestation who rapidly deteriorated after delivery due to cardiopulmonary insufficiency, dying on day one of life. Postmortem examination confirmed a non-compacted hypertrophic left ventricle but assessment of monolysocardiolipin and cardiolipin levels excluded a diagnosis of Barth syndrome. Biochemical analysis of his muscle biopsy revealed evidence of a marked isolated complex II deficiency. Sequencing the genes involved in succinate dehydrogenase structure and assembly was undertaken and revealed a novel homozygous c.275A>G, p.(Asp92Gly) *SDHD* mutation which was shown to be recessively inherited through segregation studies.

Patients with an isolated complex II deficiency harbour either compound heterozygous or homozygous mutations in an SDH structural or assembly factor gene. The resultant loss of OXPHOS-driven ATP synthesis is associated with clinical presentations including Leigh syndrome, cardiomyopathy and leukodystrophy, that often present during infancy, though adult cases are reported (Taylor et al. 1996; Birch-Machin et al. 2000). Complex II deficiency is very rare, perhaps reflecting an incompatibility with life for many cases and our patient’s clinical history with prenatal cardiomyopathy and rapid deterioration postpartum supports this hypothesis. Although the published cohort of patients with complex II deficiency is small, mutations which affect the ability of complex II to bind to the mitochondrial membrane are evolving to be the most deleterious.

The only other *SDHD*-deficient patient reported in the literature harboured compound heterozygous variants, one missense and one that extended the protein by three amino acids (Jackson et al. 2014). The clinical presentation of this individual differed from that of our patient who presented in utero with a cardiomyopathy that was incompatible with life. The previously described case was delivered at term after a normal pregnancy and presented at age 3 months with developmental regression following a viral infection with progressive neurological deterioration (epileptic seizures, ataxia, dystonia and continuous intractable myoclonic movements) and died at the age of 10 years. The patient described by Jackson et al. also had comparably low levels of SDHD protein on Western blot with greatly reduced levels of fully assembled complex II; a residual level of complex activity is therefore unlikely to account for the difference in presentation. We hypothesised that the p.(Asp92Gly) variant might have caused a conformational change given the location of the conserved acidic p.Asp92 residue at the N-terminus of one of the protein’s helical domains. With this in mind, we modelled the predicted impact of the patient’s p.(Asp92Gly) *SDHD* mutation on tertiary structure using in silico methodologies. Contrary to our expectations, neither I-TASSER nor PSIPRED predicted gross tertiary structural anomalies due to the substitution, despite being situated between two conserved cysteine residues; the pathogenicity is therefore assumed to lie in the nature of the amino acid properties as opposed to consequential protein misfolding. The location of the p.Asp92 residue at the helical N-termini may explain the discrepancy between the predicted Grantham scores and the functional data obtained following yeast modelling; leucine–tyrosine interactions are reported to act as stabilisers within alpha helices (Padmanabhan and Baldwin 1994) meaning the p.Asp92Tyr substitution (with a higher GD score) may therefore be less deleterious than the p.(Asp92Gly) substitution harboured by our patient. Moreover, the location of the substitution may also be important in capping the positive helical dipole, and replacement with a non-polar residue such as glycine would fail to provide the same charge stabilization. There was slight discordance between the helix predictions from I-TASSER and PSIPRED (Fig. 2b, c) but on closer inspection of the discordant residues, there was low confidence in the predictions.

Mutations in *SDHD* and other *SDHx* genes have been implicated not only in primary metabolic dysfunction, but also as drivers of neoplastic transformation in various tumour types. There is a wealth of information in the literature describing the involvement of *SDHx* gene mutations in cases of hereditary and sporadic cancers including head and neck paraganglioma, pheochromocytoma and gastrointestinal stromal tumours (Miettinen and Lasota 2014). In the context of hereditary cancer, each somatic cell harbours one heterozygous germline mutation either inherited from a parent or occurring *de novo.* This single loss-of-function allele, alone, is insufficient to cause neoplastic transformation but if a “second hit” affects the wild-type allele, the loss of SDH activity disrupts ATP production. The inability of SDH to metabolise succinate causes a build-up of substrate, with elevated succinate levels stabilizing HIF1α. This in turn creates a pseudo-hypoxic state, prompting a switch to glycolytic respiration consistent with neoplasia (Hanahan and Weinberg 2011; Pollard et al. 2005). The metabolic stalling due to SDH dysfunction also acts to inhibit multiple 2-oxoglutarate-dependent histone and DNA demethylase enzymes resulting in widespread histone and DNA methylation, further adding to the tumorigenic burden of these already respiratory-deficient cells (Xiao et al. 2012).

To date, mutations reported in the SDHx genes are loss of function, either as tumour suppressors or in metabolic enzymes. The mutation harboured by our patient transcends these fields in that, although manifesting as a primary metabolic condition in our case, the p.Asp92 residue is recognised as a Dutch founder HNPGL mutation, p.(Asp92Tyr). Given the established link between tumorigenesis and this residue, further functional investigations were undertaken to determine whether the p.(Asp92Gly) variant—associated with primary metabolic dysfunction—was as deleterious, or indeed more so, than the founder HNPGL mutation. Functional investigations were supportive of a deleterious effect, Western blotting of patient muscle homogenates revealed a reduction in the steady-state levels evident for not only SDHD, but also for SDHA. This was supported by one-directional BN-PAGE, which confirmed a decrease in fully assembled complex II, consistent with the hypothesis that an inability to anchor the unstable complex within the mitochondrial membrane triggers the recycling of intermediates to prevent aggregation. This turnover is seen in other cases of mitochondrial complex dysfunction and prevents accumulation and aggregation of assembly intermediates and surplus complex subunits (Alston et al. 2012).

To assess the pathogenic role of the novel p.(Asp92Gly) SDHD substitution, we carried out a series of experiments in yeast devoid of *SDH4*, the yeast *SDHD* orthologue. The use of ethanol or glucose as a carbon source tested the strains’ ability to rely upon either OXPHOS or fermentation for ATP synthesis. The SDHD residue p.Asp92 shows high evolutionary conservation and corresponds to p.Asp98 in yeast. Given that a germline mutation involving the same amino acid has been reported as a cause of paraganglioma [p.(Asp92Tyr)], two mutant alleles—*sdh4*^D98G^ and *sdh4*^D98Y^—were constructed to compare the phenotypes associated with the different substitutions. Consistent with the reduction of SDHD steady-state levels and fully assembled complex II found in our patient, the p.(Asp92Gly) mutation was detrimental to both oxidative growth and succinate dehydrogenase activity in yeast. Contrariwise, the p.(Asp98Tyr) HNPGL-associated substitution did not affect oxidative growth and showed a mild, albeit significant, reduction of SDH activity. Altogether the results obtained in the yeast model provide compelling functional evidence supporting the pathogenic role of the p.(Asp92Gly) mutation and show that this substitution conveys a more severe phenotype than the founder HNPGL *SDHD* mutation, this finding is not unique as other PGL-associated *SDHD* mutations were found to cause a milder phenotype when modelled in yeast (Panizza et al. 2013). Whilst our modelling suggests that the well-characterised p.(Asp92Tyr) *PGL* mutation is associated with what might be considered a mild phenotype in yeast, the phenotype in question is not a primary metabolic one and indeed it is only associated with oncogenesis in tandem with a second mutation, which is often a large-scale deletion or other null allele.

There were no reports of potential *SDHD*-associated cancers in the immediate family although further information from extended family members was unavailable. We previously reported inherited recessive *SDHB* mutations in association with a paediatric primary mitochondrial phenotype and this case also lacked a history of hereditary cancer (Alston et al. 2012). It is unclear whether germline carriers of the p.(Asp92Gly) *SDHD* mutation are at elevated risk of HNPGL and despite no tumours having been reported in the family, it is the opinion of their clinicians that surveillance was advisable and is ongoing.

Left ventricular non-compaction is a rare form of cardiomyopathy characterised by abnormal trabeculations in the left ventricle and associated with either ventricular hypertrophy or dilation. In some patients, LVNC arises from a failure to complete the final stage of myocardial morphogenesis, but this is not a satisfactory explanation for all cases, particularly those associated with congenital heart defects or arrhythmias. LVNC is genetically heterogeneous with many cases remaining genetically undiagnosed, but metabolic derangements are common and this form of cardiomyopathy is typical of Barth Syndrome, a disorder of mitochondrial cardiolipin typically accompanied by neutropenia (Chen et al. 2002) and has also been observed in other mitochondrial disorders including those due to mutations in mtDNA (Pignatelli et al. 2003).

In conclusion, our case further expands the clinical and genetic heterogeneity associated with isolated complex II deficiency and demonstrates that sequencing analysis of all SDH subunits and assembly factors should be undertaken for patients in whom an isolated succinate dehydrogenase defect has been identified.
